# Antimicrobial and antibiofilm activities of culture filtrates from *Lactiplantibacillus plantarum* isolated from traditional dairy products in Menoufia, Egypt

**DOI:** 10.1186/s12866-025-04404-7

**Published:** 2025-11-15

**Authors:** Mohamed T. Shaaban, Fatma Omar Khalil, Eman Hamdy, Amany A.M. Ahmed

**Affiliations:** 1 Botany and Microbiology Department, Menoufia University, Shebin El-Kom, Egypt; 2 Clinical Microbiology and Immunology Department, National Liver Institute- Menoufia University, Shebin El-kom, Menoufia Egypt

**Keywords:** Lactic acid bacteria, Antibiofilm activity, Pathogenic bacteria, Chemical profiling, ELISA reader, SEM, TEM

## Abstract

**Background:**

The global rise in multidrug-resistant (MDR) bacterial infections poses a serious threat to public health, largely driven by the overuse and misuse of antibiotics. In this context, lactic acid bacteria (LAB) and their postbiotic metabolites have attracted attention for their natural antimicrobial and antibiofilm activities. This study aimed to evaluate the effects of LAB-derived postbiotics against four clinically relevant pathogens: *Escherichia coli ATCC 25,922*,* Staphylococcus aureus ATCC 6538*,* Pseudomonas aeruginosa* ATCC 9027, and a clinical isolate of *Klebsiella pneumoniae.*

**Results:**

LAB strains were isolated from traditional fermented dairy products in Menoufia, Egypt, including Kareish cheese, Rayeb milk, and local yoghurt, using MRS agar medium. Among twelve active isolates, strain EH1 demonstrated the highest inhibitory activity. Identification via VITEK 2 and 16 S rRNA sequencing confirmed the isolate as *Lactiplantibacillus plantarum*. Optimal culture conditions for EH1 included 48 h incubation at 37 °C, pH 7.0, with yeast extract and sucrose as the preferred nitrogen and carbon sources, respectively. Antibiofilm activity was confirmed through Congo red agar assay, agar well diffusion, ELISA-based quantification, and visualization by SEM and TEM, all indicating substantial disruption of biofilm structure and cell morphology.

**Conclusions:**

Postbiotics produced by *L. plantarum* exhibited strong antibacterial and antibiofilm activity against resistant bacterial strains. These results highlight the promising potential of LAB-derived metabolites as natural, safe, and effective alternatives for inhibiting biofilm formation and mitigating the growing threat of antibiotic resistance.

**Supplementary Information:**

The online version contains supplementary material available at 10.1186/s12866-025-04404-7.

## Introduction

 Emergence of antibiotic-resistant microorganisms has emerged as one of the biggest threats to global health [1, 2]. The World Health Organization (WHO) claims that bacteria resistant to antibiotics are responsible for the spread of most infections, including gonorrhea, foodborne illnesses, TB, and blood poisoning [3, 4]. Antibiotic resistance, the host’s immune system, and other outside factors have made biofilm a global health concern. The development of biofilms by harmful bacteria is the reason why antibiotics are becoming less effective in treating illnesses. With polysaccharides, nucleic acids, and other biological materials encased in an extracellular matrix, biofilms are intricate, frequently polymicrobial colonies of microorganisms that have stuck to a surface. Because the minimum inhibitory concentrations needed to treat biofilms are frequently hundreds of times higher than those needed for planktonic cells, this matrix increases the biofilm’s resistance to antimicrobial treatments [5, 6]. Formation of biofilm is a major determinant factor in bacterial infection development [7]. Biofilms are encased in a matrix of extracellular polymers and stick together on surfaces. Biofilm bacteria are up to 1000 times more resistant to host immune responses and common drugs than plankton bacteria. Numerous biological benefits, including high infectivity, drug resistance, and robust vitality, are offered to bacteria by biofilms [8, 9]. The rising rate of antibiotic resistance has prompted scientists to look for natural alternatives by using a variety of substances with antibacterial properties, in which probiotics like lactic acid bacteria have drawn more attention. According to the [10], probiotics are “live microorganisms which when provided in appropriate proportions impart a health effect on the host.”

Lactic acid bacteria (LAB) are a group of catalase-negative, Gram-positive microorganisms that do not produce spores and typically exhibit either coccoid or rod-like shapes. These bacteria are widely employed in the fermentation industry, where they contribute to the enhancement of organoleptic properties such as flavor and texture in a variety of food and animal feed products. Their functional role in fermentation processes has made them essential to the development of traditional and modern fermented goods [11]. For many years ago and until now, lactic acid bacteria have been used as probiotics. These probiotic bacteria are applied in the medical field to stimulate the immune system, improve digestion, remediate lactose intolerance, reduce cholesterol levels, and reduce allergy [12, 13].Their high nutrient load, which includes carbs, lipids, casein, proteins, vitamins, and minerals, They produce a wide range of bioactive substances during growth and fermentation that contribute to their antagonistic effects against pathogens and biofilms [14]. These effects may include membrane disruption, interference with metabolic activity, and inhibition of pathogen adhesion. LAB has several applications in the food, agricultural, and medical sectors and is categorized as GRAS (Generally Recognized as Safe) by the Food and Drug Administration [15]. They are now perfect for commercial development and are safe for both human and animal consumption [16, 17].

LAB has been classified by the Food and Drug Administration as GRAS (Generally Recognized as Safe) and has a variety of uses in the food, agriculture, and medical industries [15] Both humans and animals may safely eat them, and they are now ripe for commercial development [18].

Temperature, pH, and medium composition are some of the culture variables that might affect LAB growth and bacteriocin production. The cost of bacteriocins is one of the things that prevents their widespread usage in food preservation [19]. The duration of incubation environment, pH, temperature, and microbial growth phase are some of the culture parameters that could impact LAB’s bacteriocin production and bacterial growth [20–24].

The purposes of this research were to extract LAB from Menoufia’s artisanal milk cheese and identify the ideal growth parameters, including temperature, starting pH, and various carbon and nitrogen sources for both LAB growth and bacteriocins production to investigate their antimicrobial and antibiofilm activities.

## Materials and methods

### Materials

The media employed throughout this study included De Man–Rogosa–Sharpe (MRS) agar and broth, nutrient agar and broth, modified Congo red agar (CRA), and Trypticase Soy Broth (TSB).

### Dairy sample collection

A total of 40 traditionally fermented dairy samples (including buttermilk, yogurt, Kareish cheese, and Rayeb milk) were collected from households and local markets in Menoufia Governorate, Egypt [25]. Samples were transferred aseptically into sterile containers, properly labeled, and stored at refrigeration temperature until they were transported to the laboratory, where they were immediately processed for microbiological examination.

### Isolation of lactic acid bacteria (LAB)

LAB strains were isolated using MRS agar as a selective medium [26, 27]. Solid and liquid samples (1 g or 1 mL) were suspended in 9 mL of sterile distilled water and homogenized. After 10 min of settling, serial dilutions were prepared, and 0.5 mL of each dilution was spread onto MRS agar plates. The plates were incubated aerobically at 37 °C for 48 h. Colonies with distinctive morphology were purified by sub-culturing and stored in MRS broth with 10% glycerol at − 20 °C.

### Detection of biofilm formation

#### Selection of bacterial strains

Three standard strains—*Escherichia coli* ATCC 25,922, *Staphylococcus aureus* ATCC 6538, and *Pseudomonas aeruginosa* ATCC 9027—were obtained from the Department of Microbiology, Faculty of Science, Menoufia University. Additionally, clinical isolates of *Klebsiella pneumoniae*, *E. coli*, *S. aureus*, and *P. aeruginosa* were obtained from agricultural sources via the National Liver Institute, Shebin El-Kom.

##### (a) tube method (Qualitative)

The qualitative detection of biofilm was conducted using the tube method as described in [28]. Each bacterial strain was inoculated into TSB with 1% glucose and incubated at 37 °C for 24 h. Tubes were emptied, washed with PBS (pH 7.3), air-dried, and stained with 0.1% crystal violet. Excess stain was removed with distilled water, and tubes were inverted for drying.

##### (b) congo red agar (CRA) method

The CRA method was used to assess biofilm-forming ability based on the method in [29–31]. The medium was prepared using brain heart infusion broth, 0.8% Congo red, and 20 g/L agar. It was autoclaved at 121 °C for 15 min. Bacterial strains were streaked and incubated at 37 °C for 24 h. Biofilm producers were identified by black, dry, crystalline colonies.

##### (c) microtiter plate assay (Quantitative)

Biofilm quantification was performed using 96-well polystyrene microtiter plates as per [32]. Overnight cultures adjusted to 0.5 McFarland in TSB with 0.25% glucose were added (200 µL per well) and incubated at 37 °C for 48 h. After removing planktonic cells, wells were fixed with methanol, stained with 2% crystal violet, washed, and then treated with 200 µL of 33% acetic acid. OD was measured at 540 nm using an ELISA reader. Biofilm strength was categorized based on OD values. All experiments were done in triplicate.

### Antibiofilm activity of LAB isolates

LAB culture supernatants were tested for antibiofilm activity using Congo Red agar plates.pathogenic Bacterial inoculums were spread plated on CRA. Next, We do discs in CRA with tibs then injected empty discs to saturate with different concentration of LAB supernatants adjusted to 0.5 McFarland were placed on the plates.Plates were incubated at 37 °C for 24–48 h, and inhibition zones were measured in millimeters.

### Identification of active LAB isolate

The LAB isolate with the strongest activity was subjected to phenotypic and genotypic identification. Colony morphology, Gram staining, and cell shape were evaluated under a microscope. Catalase activity was checked using 3% hydrogen peroxide [33–35]. VITEK 2 system was used for biochemical identification at the Liver Institute [36, 37].

16 S rRNA sequencing was done using primers 27 F and 1492R following [38], and sequencing was performed at Macrogen (South Korea). Sequences were submitted to GenBank, and a phylogenetic tree was constructed for confirmation [39].

### Optimization of LAB growth and bacteriocin production

Growth conditions were optimized in MRS broth with different incubation periods (24, 48, 72 h), temperatures (20, 30, 37, 50 °C), pH levels (3–10), carbon sources (glucose, lactose, starch, sucrose, fructose), and nitrogen sources (peptone, yeast extract, meat extract, urea, ammonium chloride, ammonium nitrate) as per [40].

###  Antibacterial activity of LAB supernatants

The antibacterial activity was tested against standard and clinical strains using the agar well diffusion method [41]. Nutrient agar plates were inoculated, and wells were filled with 100 µL of LAB supernatant. After 24 h at 37 °C, inhibition zones were measured.

### Quantitative assessment of antibiofilm activity

The inhibition of biofilm formation by LAB supernatants was also evaluated in 96-well microplates. Test wells received 100 µL of bacterial culture and 50 or 100 µL of LAB filtrate. Controls included TSB and blank wells. After 16–24 h at 37 °C, wells were stained with 1% crystal violet, and absorbance at 570 nm was recorded. The inhibition rate was calculated as:

$$\mathrm{Biofilm}\;\mathrm{inhibition}\;(\%)\;=\;1-\left({\mathrm{OD}}_{570}\;\mathrm{with}\;\mathrm{LAB}/{\mathrm{OD}}_{570}\;\mathrm{without}\;\mathrm{LAB}\right)$$

### Electron microscopy (SEM and TEM)

SEM and TEM were used to examine ultrastructural effects on treated vs. untreated bacterial cells [42]. Bacteria were fixed in 1:1 paraformaldehyde: glutaraldehyde solution for 1 h. Imaging was conducted at Alexandria University using JEOL JSM-1400 PLUS.

### Statistical analysis

All assays were done in triplicate. Data were reported as mean ± SE. One-way ANOVA was conducted using SPSS (version XX), with *p* < 0.05 as the significance threshold. Tukey’s HSD test was used for post-hoc analysis using the Agricola package in R. Statistically distinct groups were labeled with different letters.

## Results

###  LAB strain isolation and identification

**Fig. 1:**
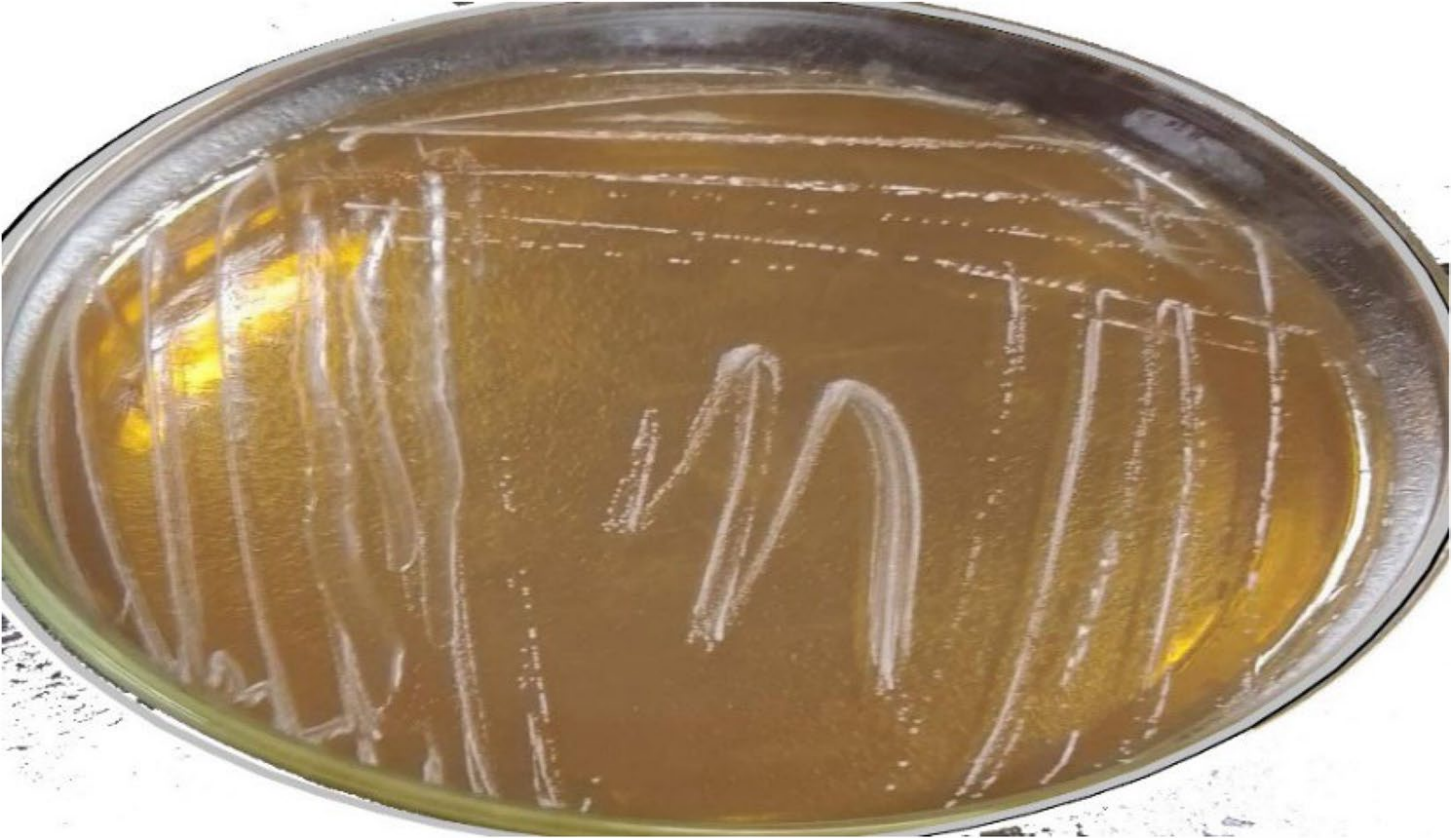
purified colonies of lactic acid bacteria on MRS Agar media

A total of twelve isolates were selected from various dairy sources, including raw buffalo milk, whey, quraish cheese, white yogurt, cream cheese, white cheese, and rayeb milk, based on colony morphology, cell shape, and staining characteristics. All selected isolates were Gram-positive, non-motile, non-spore-producing, and catalase-negative. They produced distinctive small, whitish-creamy colonies on MRS agar (Fig. 1). Due to their ability to grow under anaerobic conditions, these isolates were designated as LAB-1 to LAB-12, indicating their affiliation with lactic acid bacteria.

#### Colony description

The colonies were moderate in size, white, shiny, smooth, entire, raised, and round.

#### Gram staining

**Fig. 2:**
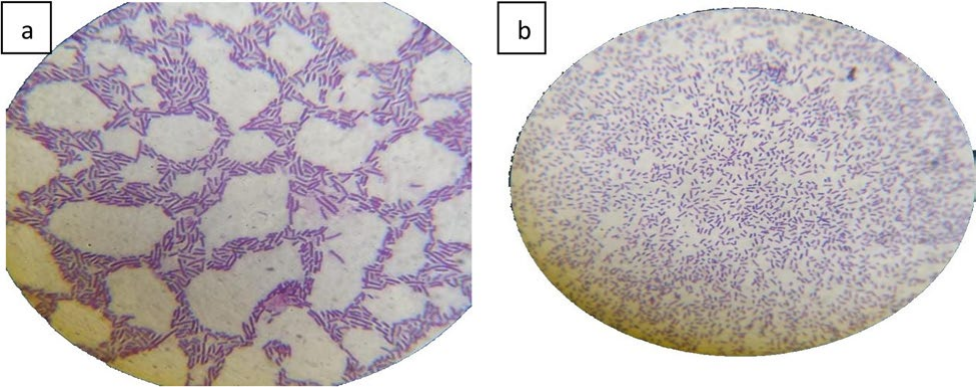
Gram stain of isolated bacteria. **a**: strepto bacilli, **b**: long mono and diplo bacilli

The *Lactiplantibacillus* isolate stained Gram-positive, demonstrating a strong cell wall that retains the purple stain. The bacteria appeared as long rod-shaped cells, occurring singly or in pairs (Fig. 2).

### Methods for detection of biofilm

The reference strains (*Pseudomonas aeruginosa* ATCC 9027, *Staphylococcus aureus ATCC 6 538*, and *Escherichia coli* ATCC 25922) were used for antimicrobial susceptibility testing in accordance with CLSI guidelines. Additionally, clinical isolates of *Escherichia coli*, *Pseudomonas aeruginosa*, *Staphylococcus aureus*, and *Klebsiella pneumoniae* were biochemically identified and subjected to antimicrobial susceptibility testing using the VITEK 2 automated system (BioMérieux, France) in table (S1 and S2).

#### a) tube method (TM)

**Fig. 3:**
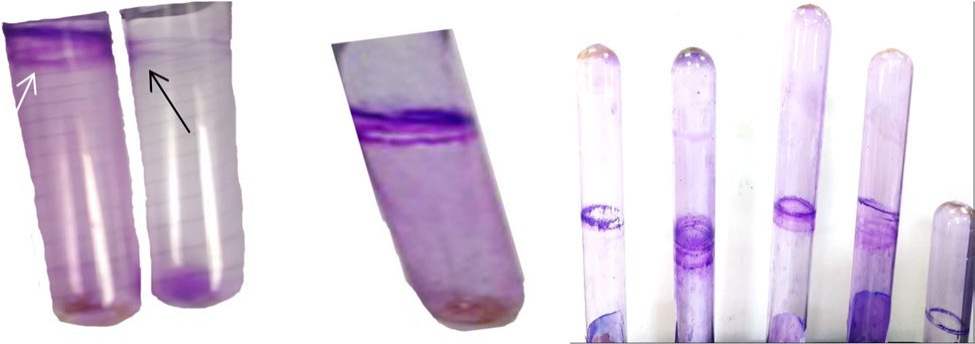
Tube adherance method (TM). White arrow refer to a violet thick film lined the wall of the tube indicates positive biofilm production and black arrow indicate lacking of biofilm production

The tube adherence method (TM) is a qualitative assay used to detect biofilm-producing microorganisms. A thick film lining the tube walls and bottom, stained with crystal violet, indicates biofilm production. Weak or no biofilm production is inferred from absence or faint staining (Fig. 3). Although simple and cost-effective, the TM is less sensitive than other methods such as the microtiter plate assay, which allows more precise quantification of biofilm mass.

#### b) congo red agar (CRA) method

**Fig. 4:**
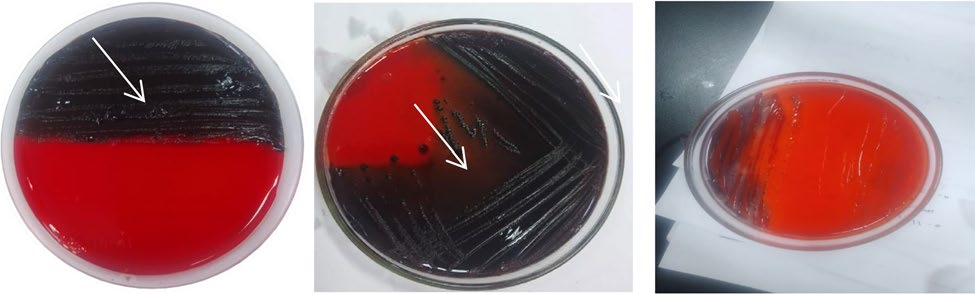
CRA method applied on CRA medium. White arrow refer to Black crystalline colonies of biofilm producer cell and pinkish-red colonies of biofilm nonproducer cell

The Congo Red Agar (CRA) method is a qualitative assay for detecting biofilm-producing microorganisms. Bacterial cultures were incubated on CRA plates for 24 to 48 h at 37 °C. Strains capable of biofilm formation secrete extracellular polysaccharides that bind to the Congo red dye, resulting in dark red or black colonies. Conversely, weak or non-biofilm producers produce red or pink colonies (Fig. 4). Although this technique allows visual identification of biofilm producers, it may fail to detect weak biofilm formers and is less sensitive than quantitative methods such as the microtiter plate assay.

#### c) biofilm phenotypic characterization using the microtiter plate test

**Fig. 5:**
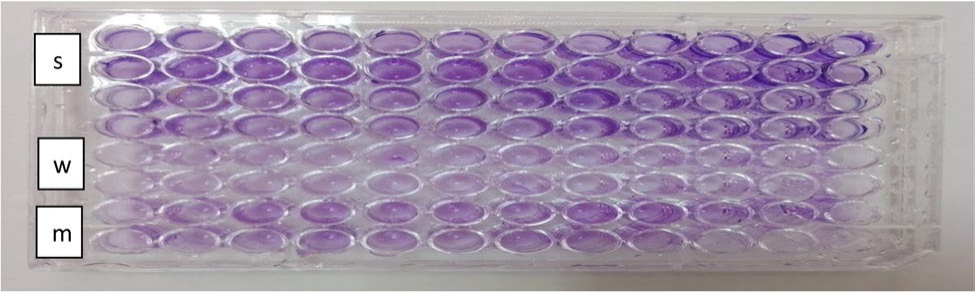
Microtiter plate assay indicating biofilm production.This result shows that the dark violet color indicates the production of biofilm pathogen bacteria (s) strong, (w) weak and (m) moderate biofilm producers in96-wells Microtiter plate

This method quantitatively detects biofilm formation using a microplate reader (micro ELISA). Results indicated that the microtiter plate (MTP) assay exhibited superior sensitivity (100%) in detecting biofilm-positive strains compared to the CRA and tube methods. The MTP assay quantitatively measures optical density of biofilms adhered to wells, providing accurate and objective results (Fig. 5). While the TM and CRA methods yielded inconsistent and sometimes contradictory findings, the MTP assay detected biofilm formation in 13 out of 15 isolates (86.7%) in this study. Notably, some strains (e.g., *E. coli* 1 and *K. pneumoniae* 2) that were negative in TM or CRA assays were identified as strong biofilm producers by MTP, demonstrating its higher sensitivity in detecting weak and moderate biofilm formers.

The antibiofilm activity of twelve *Lactiplantibacillus plantarum* isolates (L1–L12) was evaluated using the disk-diffusion method on Congo Red Agar (CRA). This medium allows for the visual detection of biofilm formation due to the interaction of Congo red dye with the exopolysaccharide matrix.

To evaluate the antibiofilm activity of the LAB isolates, culture filtrates obtained from well-grown strains were carefully introduced into wells previously made in CRA plates inoculated with pathogenic bacteria. Following incubation, the diameters of the inhibition zones surrounding the wells were measured in millimeters as an indication of the inhibitory effect.

All LAB isolates showed varying degrees of inhibition against the tested pathogens (*Staphylococcus aureus*,* Escherichia coli*,* Klebsiella pneumoniae*,* and Pseudomonas aeruginosa).* Isolate L1 showed the highest antibiofilm activity, with inhibition zones of 32.33 mm against *S. aureus* and 35.33 mm against *E. coli are presented in* Table 1*.*

**Table 1:** Antibiofilm activity of LAB isolates (L1–L12) against pathogenic bacterial strains as measured by Inhibition zones (mm) on congo red agar

Strains	Inhibition zone diameter (mm)						
	S. aureus ATCC6538	*P*. aeruginosa ATCC9027	E. coli ATCC25922	K. pneumonia	*P*. aeruginosa	S. aureus	E. coli
L1	32.33 ± 1.53 a	27.00 ± 1.73 a	22.33 ± 2.52 a	25.67 ± 1.15 a	18.67 ± 1.15 a	26.00 ± 1.00 a	35.33 ± 2.52 a
L2	28.00 ± 2.65 ab	25.67 ± 1.15 a	19.00 ± 1.73 abc	19.00 ± 1.73 b	20.33 ± 0.58 a	23.33 ± 2.89 ab	26.00 ± 1.73 b
L3	24.00 ± 1.73 b	17.67 ± 0.58 bcd	21.67 ± 1.53 ab	18.67 ± 1.15 b	15.33 ± 0.58 bc	21.00 ± 3.61 abc	17.33 ± 0.58 cd
L4	17.67 ± 2.08 c	19.33 ± 1.15 bc	18.00 ± 1.73 bcd	15.33 ± 1.53 bcd	10.33 ± 0.58 e	12.33 ± 0.58 ef	14.33 ± 0.58 cde
L5	15.33 ± 0.58 cde	16.33 ± 1.53 cde	19.00 ± 1.73 abc	17.00 ± 1.73 bc	13.33 ± 1.53 cde	15.33 ± 0.58 de	18.33 ± 2.52 c
L6	14.67 ± 0.58 cdef	19.67 ± 0.58 b	16.00 ± 1.73 cde	12.00 ± 0.00 def	12.33 ± 0.58 cde	13.00 ± 2.00 ef	18.67 ± 1.53 c
L7	16.33 ± 2.31 cd	13.33 ± 0.58 ef	15.00 ± 0.00 cdef	14.33 ± 1.53 cd	15.33 ± 1.53 bc	18.67 ± 1.53 bcd	12.33 ± 2.31 e
L8	15.00 ± 0.00 cdef	9.33 ± 0.58 g	12.33 ± 0.58 ef	11.67 ± 1.53 def	10.33 ± 1.15 e	16.33 ± 0.58 cde	15.33 ± 1.53 cde
L9	11.67 ± 0.58 ef	12.67 ± 0.58 f	14.33 ± 0.58 def	8.33 ± 1.53 f	11.33 ± 0.58 de	9.67 ± 0.58 f	14.33 ± 0.58 cde
L10	13.00 ± 1.73 def	14.00 ± 1.00 ef	11.33 ± 0.58 f	15.33 ± 0.58 bcd	10.33 ± 0.58 e	13.33 ± 1.15 ef	11.33 ± 0.58 e
L11	16.67 ± 1.15 cd	18.33 ± 1.53 bcd	14.67 ± 1.53 def	13.33 ± 0.58 cde	17.67 ± 1.15 ab	14.33 ± 1.15 def	13.33 ± 2.31 de
L12	10.67 ± 1.15 f	15.67 ± 1.15 def	12.67 ± 1.15 ef	10.33 ± 0.58 ef	14.33 ± 1.53 cd	12.33 ± 1.53 ef	12.33 ± 0.58 e

L2 also exhibited strong activity, while L3–L5 showed moderate effects. Isolates L6–L12 had weaker or limited inhibitory zones, with L12 showing the least activity overall. Notably, *P. aeruginosa* strains exhibited lower sensitivity, as reflected in smaller inhibition zones for most LAB isolates.

Statistical analysis indicated significant differences (*p* < 0.05) among the LAB strains, as shown by different grouping letters (a–f) in the data table (Table 1).

**Fig. 6:**
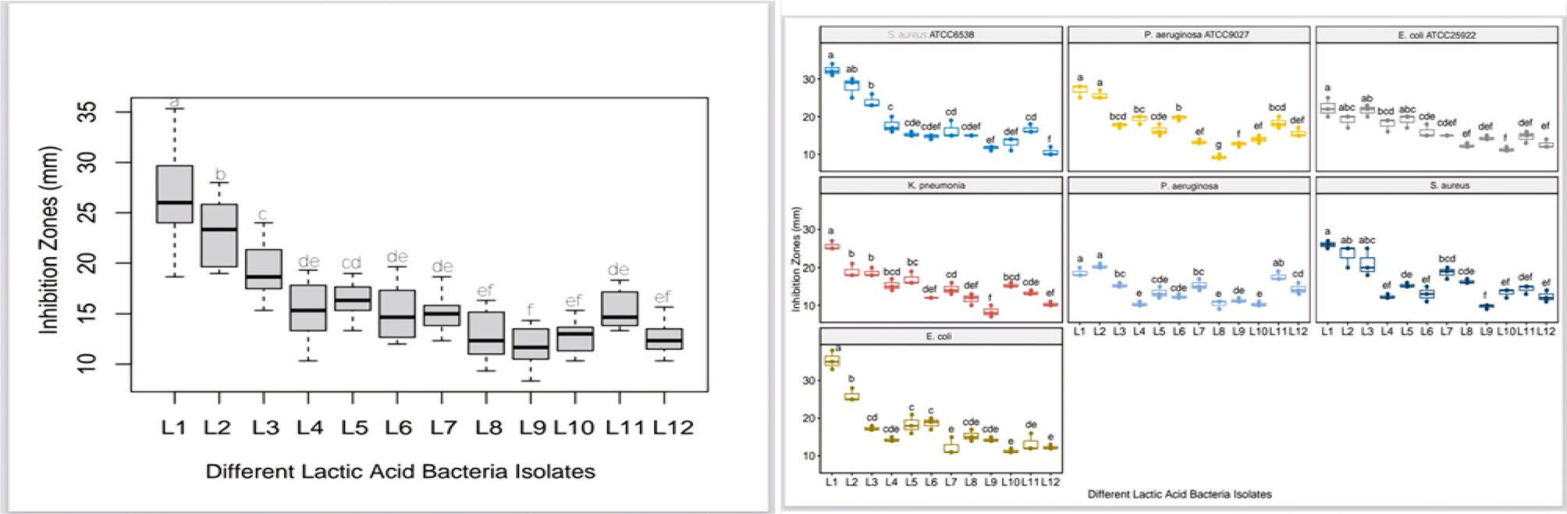
The antibiofilm activity of different *LAB* isolates (L1–L12) against pathogenic bacterial biofilms was assessed using the disk-diffusion method on Congo Red Agar. The results are presented as mean inhibition zone diameters (mm) with standard deviation (SD). Columns with different superscript letters (a, b, f) indicate statistically significant differences among the isolates (*p* < 0.001)

**Fig. 7:**
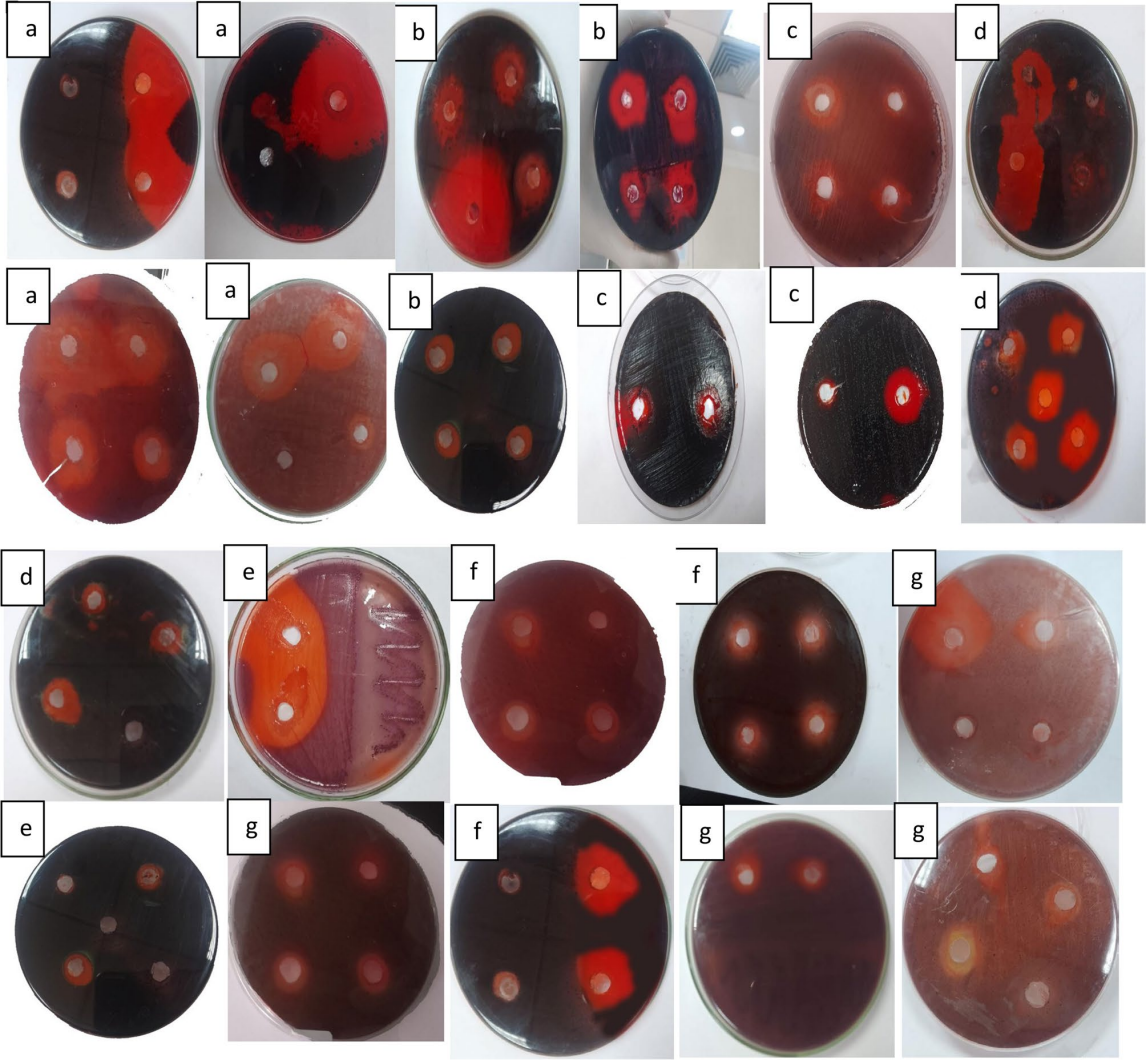
Antibiofilm produced by LAB supernatants against pathogenic bacterial biofilm by using congo red agar media, a refers to *S. aureus* ATCC6538,b refers to *P. aeruginosa* ATCC9027,c refers to *E. coli* ATCC25922, d refers to *K. pneumonia*,e refers to *P. aeruginosa.*f refers to *S. aureus* and g refers to *E. coli*

The phenotypic characterization of biofilm formation using tube method (TM), Congo red agar (CRA), and microtiter plate (MTP) methods is presented in (Table 2). Representative images showing antibiofilm activity of LAB isolates are provided in (Figs. 6 and 7).

**Table 2:** Phenotypic characterization of biofilm formation by different methods

Pathogenic bacterial isolates	Biofilm production byTM.	biofilm production by CRA.	biofilm production by MTP
*Pseudomonas aeruginosaATCC9027*	Violet ring	Black	Strong Adherence
*Staphlococcus aureus ATCC6538*	Violet ring	Black	Strong Adherence
*Escherichia coli ATCC25922*	Violet ring	Black	Strong Adherence
*Klebsiella pneumoniae 1*	Violet ring	Black	Strong Adherence
*Escherichia coli1*	Negative	Red	Strong Adherence
*Pseudomonas aeruginosa1*	Negative	Red	Weak Adherence
*Staphlococcus aureus 1*	Violet ring	Black	Strong Adherence
*Klebsiella pneumoniae2*	Negative	Red	Strong Adherence
*Escherichia coli2*	Negative	Red	Negative
*Pseudomonas aeruginosa2*	Violet ring	Black	Moderate Adherence
*Staphlococcus aureus2*	Violet ring	Red	Moderate Adherence
*Klebsiella pneumoniae3*	Violet ring	Black	Moderate Adherence
*Escherichia coli3*	Violet ring	Red	Weak Adherence
*Pseudomonas aeruginosa3*	Negative	Black	Strong Adherence
*Staphlococcus aureus3*	Violet ring	Black	Strong Adherence

a, b, …. and f: means having different superscripts within each column are significantly different (*p* < 0. 001)

*SD* Standard deviation

Biochemical characterization of LAB isolate EH1 was performed with the VITEK 2C2 microbial identification system, and the results are summarized in (Table 3). According to the manufacturer, “+” refers to positive test results and “−” refers to negative test results.

**Table 3:** Chemical characterization of LAB isolate E.H1 using the VITEK 2c2 microbial identification system (*BioMérieux*,* Craponne*,* France)*

Test	Result	Test	Result	Test	Result	Test	Result
Amy	-	TyrA	+	**Dxyl**	-	dMAL	+
APPA	-	ILATk	-	**BGAR**	-	MdG	-
LeuA	-	NC 6.5%	+	**AGAL**	+	dTRE	+
AlaA	-	O129R	+	**URE**	-	AGLU	-
dRIB	-	dXYL	-	**NAG**	+	PHOS	+
NOVO	-	AspA	-	**d MNE**	+	BGUR	-
dRAF	+	BGURr	-	**SAC**	+	dGAL	+
OPTO	+	dSOR	-	**BGAL**	+	BACL	+
PIPLC	-	LAC	+	**AMAN**	-	PUL	-
CDEX	-	dMAN	+	**PyrA**	+	ADH2s	+
ProA	-	SAL	+	**POLYB_R**	+	dMAL	+

### Optimization of culture conditions for LAB growth and bioactive filtrate production

**Fig. 8:**
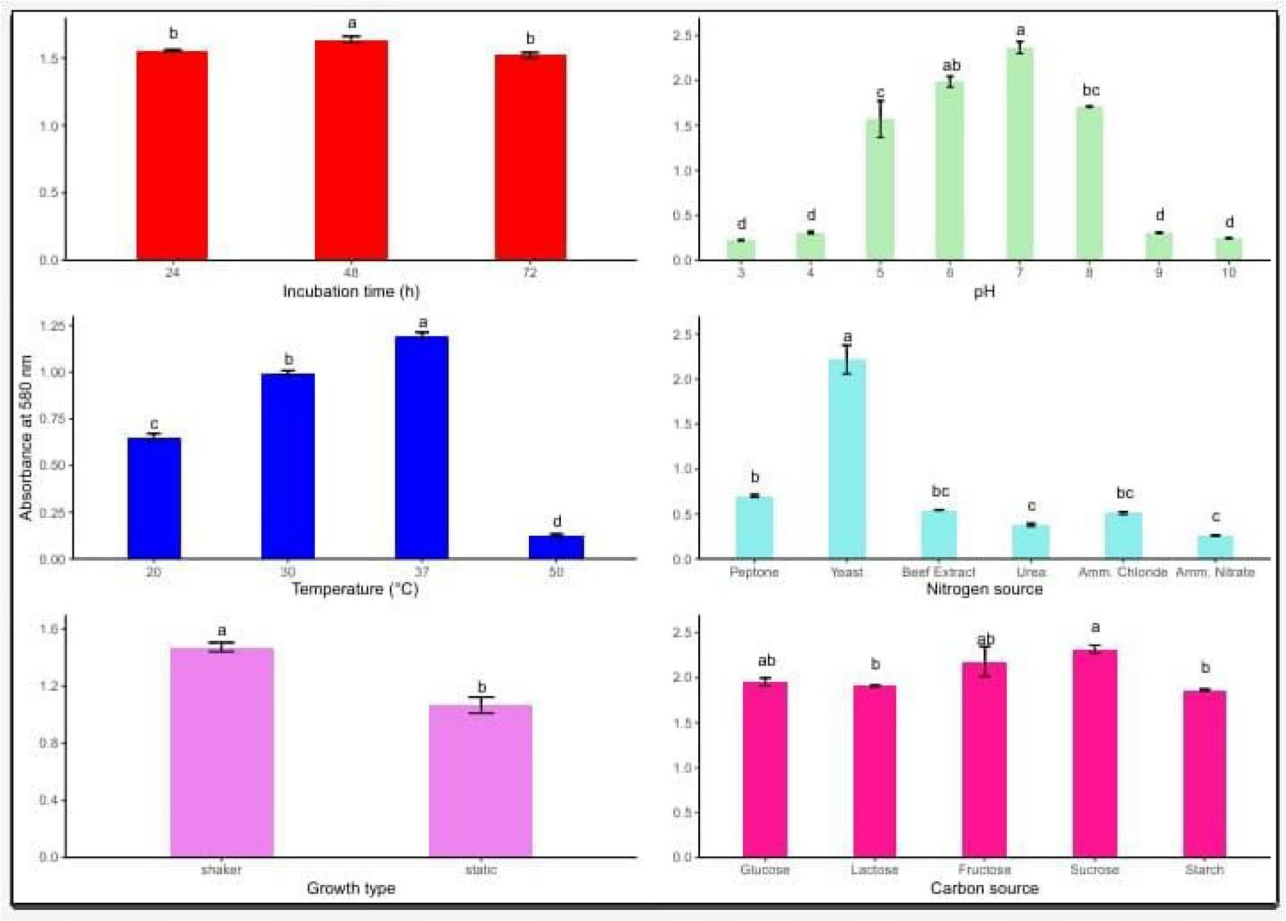
Effect of various influencing parameters on LAB growth. Values are expressed a, b. and f: means having different superscripts within each column are significantly different (*p* < 0. 001). SE standard error

The results were measured with a spectrophotometer measuring the optical density at 600 nm in (Fig. 8). *Lactiplantibacillus plantarum’*s development and bacteriocin production are heavily influenced by a number of dietary and environmental variables growth type, nitrogen and carbon sources, as well as fermentation conditions including pH, temperature, and incubation duration. To increase antibacterial action and biomass yield, these parameters must be optimized. The results show how the maximum bacteriocin activity was attained, suggesting that the mid-logarithmic phase is ideal for biosynthesis after 48 h. Shorter (24 h) or longer (72 h) incubations resulted in lower activity. With notable drops at 20 °C and 30 °C, and almost no activity at 50 °C, either due to metabolic inhibition or protein denaturation, 37 °C was the ideal temperature for both growth and bacteriocin synthesis. With the maximum activity seen at pH 6–7, pH also had a significant effect. In contrast, both acidic (pH 3–5) and alkaline (pH 8–10) circumstances caused a noticeable decrease, maybe as a result of cellular stress or enzyme deactivation. The most efficient nitrogen supply was yeast extract, perhaps because it contained a lot of peptides, amino acids, and vitamins. Peptone provided a modest amount of support, while the least effective sources were inorganic ones like urea, ammonium chloride, and ammonium nitrate. *L. plantarum* can effectively use simple sugars for energy and metabolite synthesis, as evidenced by the highest bacteriocin production from sucrose and fructose, followed by glucose and lactose, when considering carbon sources. In contrast, starch demonstrated lower effectiveness, most likely because of its complex structure that necessitates enzymatic hydrolysis. Furthermore, bacteriocin production was much increased under shaking conditions as opposed to static culture, presumably as a result of better aeration, oxygen availability, and uniform nutrient distribution. These findings highlight how crucial it is to optimize physicochemical and nutritional parameters in order to increase the effectiveness of *L. plantarum’s* capacity to produce bacteriocins, which may find use in the creation of antimicrobial agents and natural food preservation.

### Physiological and molecular identification of LAB isolates growth 

**Fig. 9:**
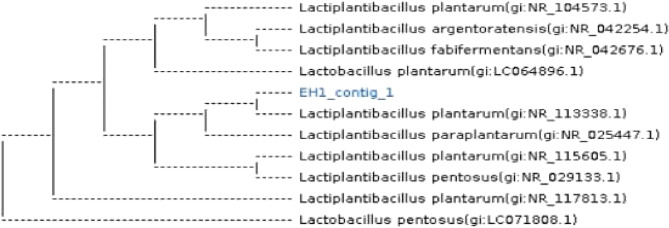
Phylogenetic tree illustrating the connection between a few chosen isolates and their Genbank homologs. MEGA6 sequence alignment software was used to build the tree for *Lactiplantibacillus plantarum* strains JCM 1149 and LC064896.1

Total genomic DNA was extracted from each of the isolate LAB-1, with the highest probiotic p otential in order to identify characteristics of their genotypic. According to **Sadrani et al. (2014)**, this extraction was carried out in order to identify the isolates using bacterial-specific 16 S rRNA amplification of gene sequencing, which enables identification of bacteria down to the genus and species level. Selected isolates’ 16 S rRNA gene sequences were compared to those found in the Gene-Bank databaseMolecular identification based on 16 S rRNA gene sequencing confirmed that the most promising isolate, LAB-1, belongs to the genus *Lactobacillus* and specifically to *Lactiplantibacillus plantarum*, showing 98.7% similarity to strains JCM 1149 and LC064896.1 (Fig. 9).

https://www.ncbi.nlm.nih.gov/nuccore/OQ538171.1/.

The bacterial strains have been recorded at the GenBank with the identification number JCM 1149, LC064896.1.

### Lactiplantibacillus plantarum strain’s antibacterial and antibiofilm properties

The antibacterial activity of LAB strains was first tested using the agar well diffusion method. *Pseudomonas aeruginosa* ATCC9027, *Staphlococcus aureus* ATCC6538, *Escherichia coli* ATCC25922, and *Klebsiella pneumoniae* were the examined bacterial strains against which the bacterial strains displayed discernible inhibitory zones. Table 4 displays the LAB strains’ inhibitory zones against each bacterial strain. They cause membrane disruption, which increases membrane permeability, causes cell lysis, causes metabolite loss, and ultimately results in the death of the bacteria. Additionally, the effect of the *L. plantarum* supernatant on the cellular ultrastructure of *S. aureus*,* P. aeruginosa and E. coli* was examined by SEM and TEM before and after treatment. The SEM and TEM micrographs revealed the control to have normal morphological characteristics (Figs. 11 and 12), displaying a smooth, undamaged cell membrane. Nevertheless, the cell membrane exhibited signs of impairment, characterized by the emergence of many holes besides completely lysed cells. Also, the deformation of the cell membrane and reduction in biofilms of LAB -treated *S. aureus*,* P. aeruginosa and E. coli* as shown in (Figs. 6 and 7) For testing the antibiofilm activity of LAB, the bacterial strains were first tested for their capability to form biofilm. Using the CRA plates, all of the tested strains showed the ability to form biofilms. These biofilms were detected through the formation of black colonies on the CRA plates (Fig. 4). Afterward, using the microtiter plate method, the antibiofilm activity of different concentrations (ranging from 50 to 100 ml) of *L. plantarum* supernatants were assessed. The *L. plantarum* supernatants were found to possess a dose-dependent capacity to eliminate the preformed biofilms of *P. aeruginosaATCC9027*,* S. aureus ATCC6538*,* E. coli ATCC25922 and K. pneumoniae.* As illustrated in (Table 4), *L. plantarum was* able to eliminate and reduce the biomass of the preformed biofilms of *bacterial pathogen* by 50 and 100 ml, respectively, after 24 h of exposure.

**Table 4:** Evaluation of antibiofilm activity of *Lactiplantibacillus plantarum* supernatant (EH1)

		Mean	±	S. D	Min.	Max.	F	*P*
*S. aureus*	Biofilm	0.665	±	0.031	0.630	0.687	1223	< 0.001**
	Inoculum Conc.1/50	0.049	±	0.002	0.048	0.051		
	Inoculum Conc.1/100	0.027	±	0.005	0.022	0.031		
	P1 = < 0.001**, P2 = < 0.001**, P3 = 0.183							
*E. coli*	Biofilm	0.653	±	0.048	0.613	0.706	475	< 0.001**
	Inoculum Conc.1/50	0.045	±	0.007	0.039	0.052		
	Inoculum Conc.1/100	0.042	±	0.004	0.038	0.045		
	P1 = < 0.001**, P2 = < 0.001**, P3 = 0.900							
*P. aeruginosa*	Biofilm	0.500	±	0.043	0.454	0.538	304	< 0.001**
	Inoculum Conc.1/50	0.047	±	0.002	0.045	0.049		
	Inoculum Conc.1/100	0.036	±	0.016	0.023	0.054		
	P1 = < 0.001**, P2 = < 0.001**, P3 = 0.627							
*K. pneumonia*	Biofilm	0.492	±	0.006	0.485	0.496	9015	< 0.001**
	Inoculum Conc.1/50	0.056	±	0.004	0.053	0.061		
	Inoculum Conc.1/100	0.034	±	0.003	0.031	0.037		
	P1 = < 0.001**, P2 = < 0.001**, P3 = 0.001*							

*: Significant difference

**: High significant difference

ANOVA test, P: P-value, P1: Biofilm vs. Inoculum Conc.1/50, P2: Biofilm vs. Inoculum Conc.1/100, P3: Inoculum Conc.1/50 vs. Inoculum Conc.1/100 Antibiofilm activity of *Lactiplantibacillus plantarum* supernatant (L1).

### Evaluation of antibiofilm activity by microtiter plate-crystal violet method by using ELISA reader (ER96) apparatus

**Fig. 10:**
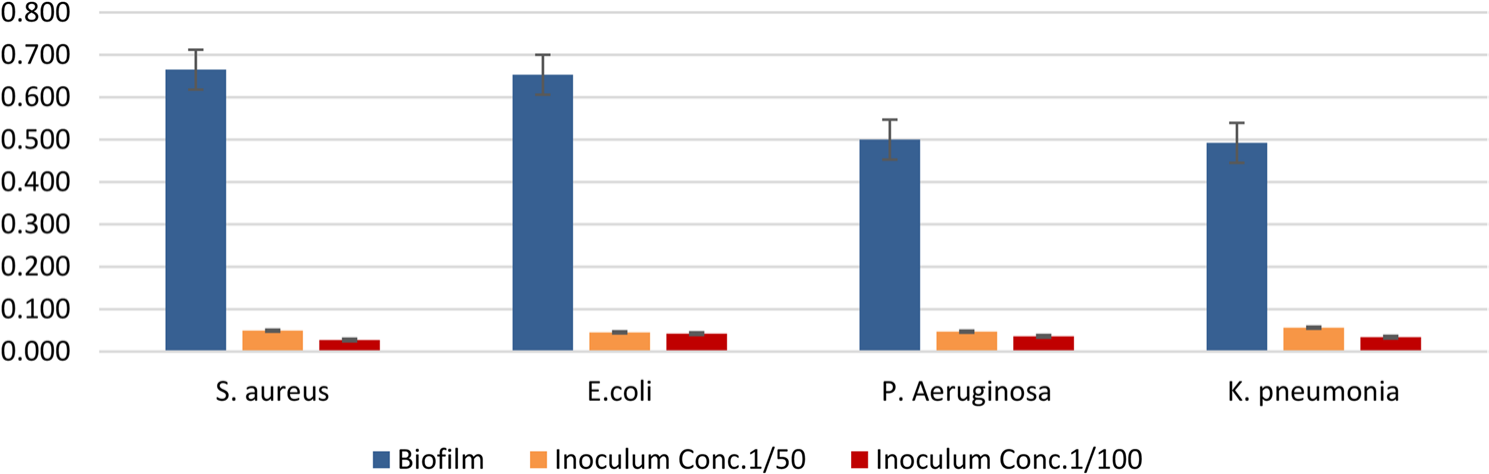
Antibiofilm activity of *Lactiplantibacillus plantarum EH1* cell-free supernatant against biofilm-forming pathogens. Biofilm formation and inhibition at two inoculum concentrations (1/50, orange; 1/100, red) were measured for *Escherichia coli*, *Staphylococcus aureus*, *Pseudomonas aeruginosa*, and *Klebsiella pneumoniae*

*Lactiplantibacillus plantarum* (EH1) antibiofilm effects were examined using the microtiter plate-crystal violet assay (Table 4 and Fig. 10)

Results are expressed as optical density (OD) values with standard error bars.

### Ultrastructural effects of cell-free filtrates of Lactiplantibacillus plantarum on biofilm-forming bacteria as observed by SEM and TEM

The effect of the cell-free supernatant (CFS) from *Lactiplantibacillus plantarum* on biofilm-forming bacteria was analyzed using scanning electron microscopy (SEM) and transmission electron microscopy (TEM). Three notable pathogenic strains—*Escherichia coli ATCC 25,922*,* Staphylococcus aureus ATCC 6538*,* and Pseudomonas aeruginosa ATCC 9027*—were chosen for this investigation because to their robust biofilm-forming characteristics.

SEM examination of untreated control samples demonstrated complete biofilms consisting of densely aggregated bacterial cells embedded within a structured extracellular polymeric substance (EPS) matrix. The bacterial surfaces exhibited a smooth and well-preserved appearance, characterized by distinct cellular boundaries and intact morphology.

Conversely, cells exposed to *L. plantarum* CFS exhibited significant morphological changes. The EPS layer appeared either collapsed or fragmented, and the bacterial cells exhibited unusual shapes with evident surface roughening, membrane perforation, and, in certain instances, complete cell collapse. The spatial arrangement of the biofilm was markedly impaired, signifying a deterioration of structural integrity.

TEM offered additional understanding of the intracellular consequences of the treatment. Untreated bacterial cells displayed uniform cytoplasm, distinct membranes, and typical internal structure. Cells subjected to the CFS exhibited significant subcellular damage. Disruption of the cytoplasmic membrane, efflux of intracellular contents, and the emergence of electron-dense granules were frequently noted. These granules may indicate aggregated or denatured proteins and nucleic acids, implying a deterioration of metabolic integrity and cellular function.

**Fig. 11:**
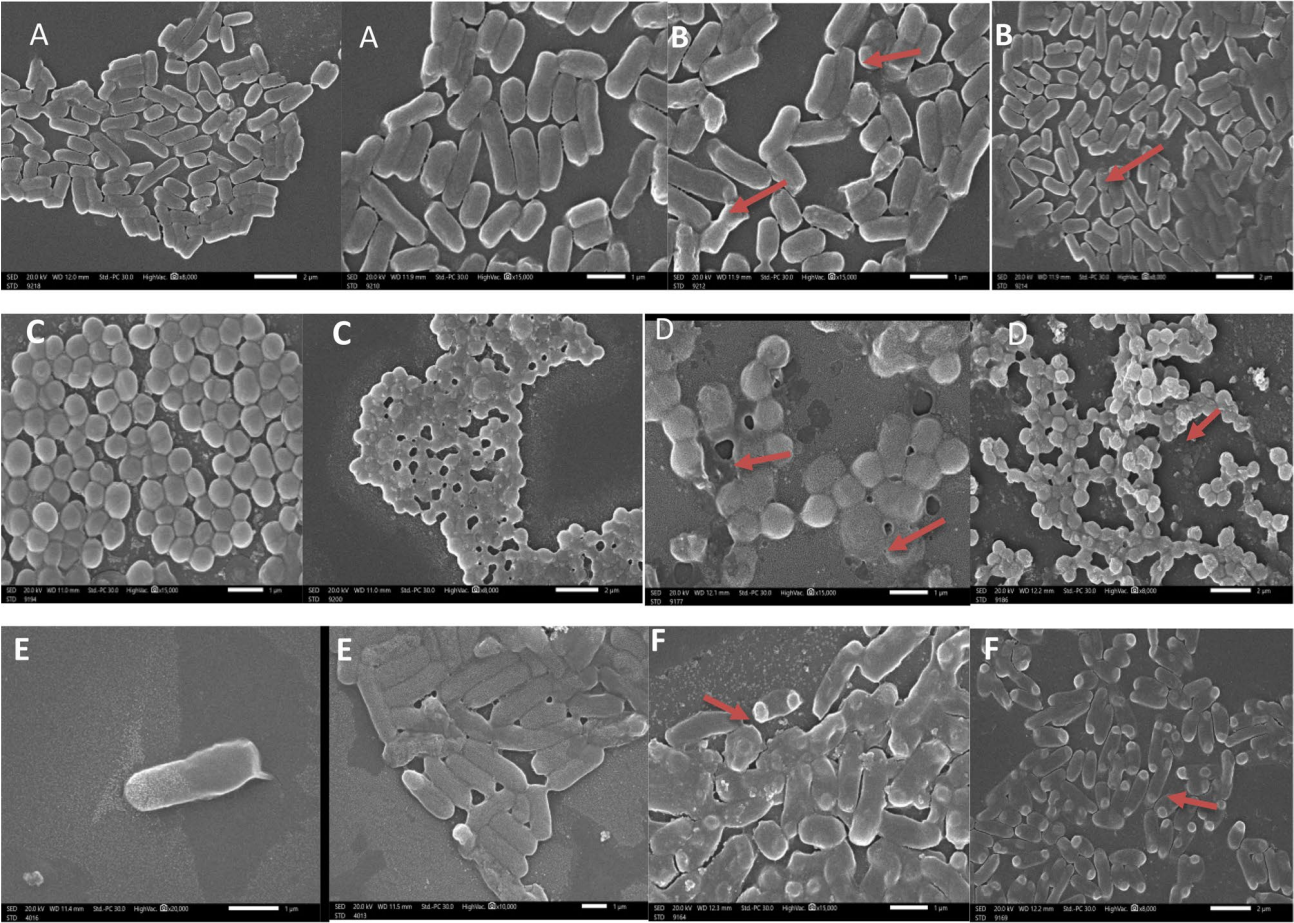
Scanning electron microscope (SEM) images showing the effect of *Lactiplantibacillus plantarum* treatment on pathogenic bacteria. **A** *Staphylococcus aureus* control cells exhibiting intact and smooth cell membranes; (**B**) S. aureus treated with *L. plantarum* showing visible deformation and damage to the cell membrane (indicated by red arrows); (**C**) *Escherichia coli* control cells with normal morphology; (**D***) E. coli* treated cells displaying membrane disruption and deformation; (**E**) *Pseudomonas aeruginosa* control cells with undamaged surfaces; (**F**) *P. aeruginosa* treated cells revealing significant membrane damage and cell surface irregularities

**Fig. 12:**
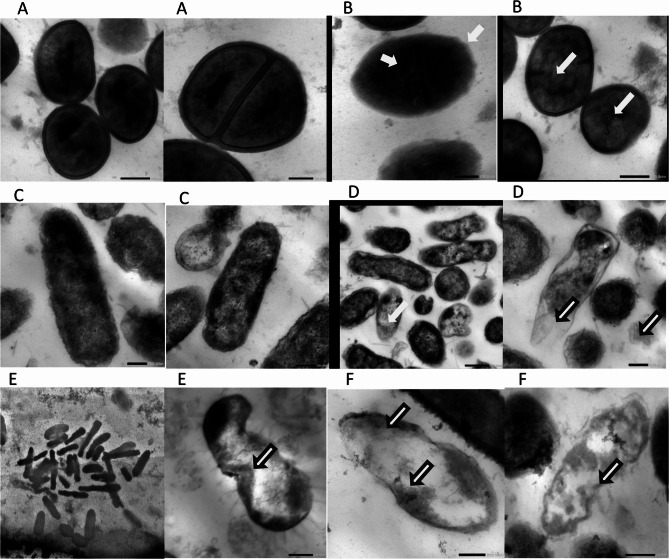
Transmission electron microscope (TEM) images showing ultrastructural changes in bacterial cells after treatment with *Lactiplantibacillus plantarum* supernatant. **A** *Staphylococcus aureus* control cells displaying normal ultrastructure; (**B***) S. aureus* treated cells showing disrupted membranes and cytoplasmic leakage; (**C**) *Escherichia coli* control cells with intact cellular structure; (**D**) *E. coli treated* cells exhibiting membrane damage and intracellular alterations; (**E**) *Pseudomonas aeruginosa* control cells with normal ultrastructure; (**F**) *P. aeruginosa* treated cells revealing membrane breakdown, cytoplasmic leakage, and electron-dense aggregates indicative of cellular damage

The SEM and TEM results collectively demonstrate that the culture filtrate has a twofold effect, disrupting both the external biofilm matrix and the interior architecture of bacterial cells. The significant distortion and failure of membrane integrity align with mechanisms that compromise cell viability and destabilize biofilms. While the specific chemical composition of the active components was not delineated in this investigation, the findings highlight the capability of these filtrates to operate as potent disruptors of mature biofilms and modify bacterial cell architecture at both surface and ultrastructural dimensions (Figs. 11 and 12).

The red arrows highlight areas of bacterial cell membrane deformation caused by the treatment.

## Discussion

The rise in bacterial antimicrobial resistance (AMR) leads to high mortality rates associated with resistant infections [1]. This alarming trend highlights the urgent need to develop novel natural compounds with antibacterial and antibiofilm properties. The present study focused on lactic acid bacteria (LAB), specifically *Lactiplantibacillus plantarum*, and their potential antibacterial and antibiofilm activities. In developing countries, biofilms pose a significant public health risk as they provide a protective environment for many microorganisms. Different species form biofilms through varied mechanisms, contributing to persistent infections in sectors such as drinking water, food and dairy industries, and water biofouling. Contaminated food and water thus represent a major source of infection threatening both human and animal health [42, 43]. Controlling biofilm formation through various biotic and abiotic parameters can effectively reduce infection risks.

LAB are gaining importance as natural alternatives to chemical preservatives and antibiotics in food technology, exhibiting inhibitory effects against gastrointestinal and uropathogens. Bacteriocins act by disrupting cell membranes, increasing permeability, inhibiting cell wall synthesis, and blocking protein and nucleic acid synthesis, ultimately leading to bacterial death [44].

In this study, *Lactiplantibacillus* species were identified phenotypically by colony morphology and Gram staining, with colonies appearing creamy-white, smooth, and medium-sized on MRS agar. The isolates were Gram-positive rods, consistent with *Lactiplantibacillus* species characteristics.

These findings align with previous reports by Zheng et al. (2020) and Darwish et al. (2023), confirming typical colony and microscopic features of *Lactiplantibacillus plantarum* [45, 46]. These morphological traits, though not definitive for species identification, serve as useful preliminary indicators.

The isolates were catalase- and oxidase-negative, further supporting their identification as LAB and facultative anaerobes, consistent with prior studies [45–47]. The genus *Lactiplantibacillus* is well known for its probiotic potential and ecological adaptability, particularly in dairy environments.

*Lactiplantibacillus plantarum* is among the most important LAB genera with widespread industrial applications. It is recognized for its probiotic benefits, including host immune modulation and therapeutic effects [46, 48]. The antimicrobial activity of LAB strains is attributed to production of metabolites such as hydrogen peroxide, organic acids, and bacteriocins, which inhibit pathogenic bacteria growth [49].

Despite these benefits, the increasing diversity of bacterial virulence factors and antimicrobial resistance mechanisms challenges the safety profile of probiotics, necessitating strain-level characterization per FAO/WHO guidelines [36, 50].

Biofilm production by bacterial isolates was assessed phenotypically using microtiter plate (MTP), tube method (TM), and Congo Red Agar (CRA) assays. The MTP assay demonstrated the highest sensitivity and specificity, confirming its status as the gold standard for biofilm detection [31]. CRA results correlated well with molecular analyses and revealed black colonies indicative of biofilm formation, consistent with prior research [51, 52].

Biochemical testing using the Vitek 2C2 system further characterized the *L. plantarum* isolates, showing positive reactions for several carbohydrate fermentation and resistance profiles, in line with previous findings [36]. Molecular identification through partial 16 S rDNA sequencing confirmed isolate EH1 as *L. plantarum* strain JCM 1149 (GenBank accession LC064896.1), corroborating the accuracy of molecular taxonomy in differentiating closely related LAB species [53].

Environmental and dietary factors are pivotal in determining the growth dynamics of *Lactiplantibacillus plantarum* and affecting the biological activity of its culture filtrates. This work systematically evaluated that the ideal incubation duration for increasing biomass and filtrate functionality was 48 h, after which a significant reduction was seen, likely due to food depletion and the buildup of metabolic wastes [54]. Temperature was a critical element; growth and filtrate activity reached their zenith at 37 °C, exhibiting reduced effects at both sub-optimal and supra-optimal temperatures, possibly indicative of enzyme inefficiency and thermal stress [55].

The initial pH substantially influenced bacterial proliferation. The strain exhibited vigorous growth at neutral pH (about 7.0), whereas acidic conditions (pH ≤ 4.0) significantly compromised viability—this aligns with previous research connecting low pH to cytoplasmic acidification and membrane instability [40, 56]. Analysis of carbon source usage indicated that simple sugars, including sucrose and glucose, facilitated the highest metabolic rates and cell density, presumably due to their rapid uptake and conversion through glycolytic pathways [57]. Yeast extract demonstrated superiority among nitrogen sources, likely due to its abundant peptides, amino acids, and necessary micronutrients, while inorganic nitrogen sources (e.g., ammonium chloride, nitrate) were comparatively less effective [58, 59].

These observations highlight the necessity of optimizing cultural factors to improve LAB output. Enhanced growth not only elevates biomass but also augments the functional characteristics of cell-free supernatants, rendering them appropriate for downstream applications [60].

There has been a significant increase in interest about probiotic-based interventions for the management of biofilm-associated illnesses, particularly concerning the contamination of medical devices such urinary and central venous catheters [61]. Lactic acid bacteria, such as Lactobacillus, Streptococcus, and Lactococcus, have been documented to provide many health benefits, including immunomodulation, gut microbiota regulation, cholesterol reduction, and allergy alleviation [12, 13].

Our work revealed a notable decrease in biofilm development following exposure to the cell-free supernatants (CFS) of L. plantarum. The effects were especially significant against robust biofilm makers like *Escherichia coli*,* Staphylococcus aureus*, and *Pseudomonas aeruginosa*, with certain strains demonstrating an almost total loss of adhesion capability [62]. The observed antibiofilm activity may result from the synergistic effects of diffusible metabolites in the filtrate, such as hydrogen peroxide, organic acids, exopolysaccharides, and potentially other unidentified compounds, although the precise mechanisms have yet to be clarified [36, 63, 64].

It is important to recognize that specific bioactive chemicals generated by LAB may preferentially influence sessile (biofilm-associated) cells while leaving their planktonic counterparts unaffected, underscoring the necessity to evaluate both bacterial stages during screening [65]. This was seen in our experimental paradigm, where biofilm biomass was markedly diminished while exhibiting negligible effects on planktonic cell survival.

To corroborate these findings, SEM and TEM examinations were conducted, yielding high-resolution images of the treated and untreated bacterial structures. In untreated samples, bacterial cells preserved their original shape and entire biofilm matrices. Conversely, cells subjected to the LAB supernatants exhibited evident surface deformation, membrane breakage, and cytoplasmic leaking. The structural integrity of the biofilm matrix was evidently disrupted, as demonstrated by EPS collapse and cellular disintegration observed through SEM and TEM imaging [66, 67]. These modifications indicate a significant disruptive impact of the filtrates on the physical structure of biofilms and the ultrastructure of bacterial cells [68].

Recent investigations underscore the significance of postbiotic metabolites in contextualizing these findings. Frontiers in Microbiology (2024) found that biosurfactants generated by *L. plantarum* markedly reduced biofilm formation and shown potent antibacterial efficacy against *S. aureus* and *E. coli* [69]. A subsequent review in Science of Food and Agriculture (2025) emphasized the utilization of LAB bioactive compounds, including postbiotics, in the control of *Salmonella* biofilms on food contact surfaces [70].

These results indicate that improving culture conditions boosts *L. plantarum* growth and improves the functional profile of its filtrates. The documented antibiofilm effects contribute to the increasing data endorsing postbiotic preparations as a viable, low-risk alternative for managing biofilm-associated infections.

This study adds to the existing knowledge by comprehensively characterizing local *Lactiplantibacillus plantarum* isolates and demonstrating their antibacterial and antibiofilm efficacy against key pathogenic bacteria. Despite promising in vitro results, limitations include the lack of detailed molecular characterization of the active antimicrobial compounds and absence of in vivo validation. Future research should focus on isolating and identifying specific bacteriocins and metabolites responsible for biofilm inhibition, investigating synergistic effects with antibiotics, and conducting animal or clinical trials to assess safety and efficacy. Addressing these gaps will enhance the practical application of *L. plantarum* as a natural biotherapeutic agent in food safety and medical fields.

## Conclusion

This study presents a novel method employing *Lactiplantibacillus plantarum* strains, extracted from traditional fermented dairy products, as a natural and sustainable source of bioactive postbiotic substances with significant antibiofilm and antibacterial properties. This research highlights the effectiveness of unpurified, cell-free culture supernatants, contrasting with other studies that mostly concentrated on pure bacteriocins or isolated metabolites, thereby providing a cost-efficient and practical option for antimicrobial applications.

The work notably combines a thorough optimization of nutritional and environmental growth parameters—specifically incubation time, pH, carbon, and nitrogen sources—to simultaneously improve bacterial proliferation and postbiotic efficacy, a dual approach that has been seldom investigated. Moreover, the utilization of transmission and scanning electron microscopy provide ultrastructural evidence of biofilm destruction and cellular injury in certain pathogens, enhancing the mechanistic understanding of the observed biological activity.

This integrated methodology offers a unique contribution to the field by facilitating the development of *L. plantarum*-derived postbiotics as environmentally sustainable agents for managing biofilm-associated infections, with potential applications in clinical therapeutics, food preservation, and pharmaceutical formulation. Subsequent investigations should focus on identifying the principal active metabolites, examining synergistic interactions with traditional antimicrobials, and assessing the efficacy of the filtrate in in vivo models. [71]

## Supplementary Information

Supplementary Material 1.

## Data Availability

“The 16S rRNA gene sequence of the isolate Lactiplantibacillus plantarum strain EH1 has been deposited in the NCBI GenBank database under accession number OQ538171.”

## Abbreviations

LAB: Lactic Acid Bacteria
CFS: Cell-Free Supernatant
MRS: de Man, Rogosa and Sharpe (agar/media)
PBS: Phosphate Buffered Saline
OD: Optical Density
ELISA: Enzyme-Linked Immunosorbent Assay
SEM: Scanning Electron Microscopy
TEM: Transmission Electron Microscopy
ATCC: American Type Culture Collection
SPSS: Statistical Package for the Social Sciences
ANOVA: Analysis of Variance
CFU: Colony Forming Unit
GRAS: Generally Recognized As Safe
CRA: Congo Red Agar
PCR: Polymerase Chain Reaction
VITEK 2: Automated Bacterial Identification System (BioMérieux)
DOI: Digital object identifier
