# Supplementary material for: Antimicrobial and antibiofilm activities of culture filtrates from Lactiplantibacillus plantarum isolated from traditional dairy products in Menoufia, Egypt
Source: BMC Microbiol. 2025 Nov 15;25:745. doi: 10.1186/s12866-025-04404-7 (PMC12619430; doi:10.1186/s12866-025-04404-7)
Supplement: Supplementary file 1 — Supplementary Material 1. [file 12866_2025_4404_MOESM1_ESM.docx]

**Results**

The reference strains (*Pseudomonas aeruginosa* ATCC 9027, *Staphylococcus aureus* ATCC 6538, and *Escherichia coli* ATCC 25922) were obtained from the Department of Microbiology, Faculty of Science, Menoufia University, Shebin El-Kom, Egypt, and were used for antimicrobial susceptibility testing in accordance with CLSI guidelines. Additionally, clinical isolates of *Escherichia coli*, *Pseudomonas aeruginosa*, *Staphylococcus aureus*, and *Klebsiella pneumoniae* were collected from farm samples provided by the Microbiology Department of the National Liver Institute, Shebin El-Kom. All bacterial isolates were biochemically identified and subjected to antimicrobial susceptibility testing using the VITEK 2 automated system (BioMérieux, France), as described by Kim et al. (2022). The antibiotic susceptibility profiles of the reference strains are summarized in (**Table 1)**, while the susceptibility profiles of clinical isolates are shown in (**Table 2)**.

# Table (1):Antibiotic Susceptibility Profile of Clinical Isolates.

| **Antibiotic (Disk Content)** | **Breakpoint (mm) (R ≤ / S ≥)** | **S. aureus ATCC6538 (mm / S-R)** | **E. coli ATCC25922 (mm / S-R)** | **P. aeruginosa ATCC9027 (mm / S-R)** |
| --- | --- | --- | --- | --- |
| **Norfloxacin (10 µg)** | ≤12 / ≥17 | 12 / R | 25 / S | 18 / S |
| **Meropenem (10 µg)** | ≤19 / ≥23 | 25 / S | 24 / S | 23 / S |
| **Amoxicillin-Clavulanic (20/10 µg)** | ≤19 / ≥20 | 21 / S | 15 / R | 22 / S |
| **Ampicillin (10 µg)** | ≤18 / ≥22 | 23 / S | 21 / S | 17 / R |
| **Nalidixic Acid (30 µg)** | ≤13 / ≥19 | - | 20 / S | 12 / R |
| **Chloramphenicol (30 µg)** | ≤12 / ≥18 | 20 / S | 18 / S | 10 / R |
| **Ciprofloxacin (5 µg)** | ≤20 / ≥31 | 31 / S | 18 / R | 32 / S |
| **Levofloxacin (5 µg)** | ≤14 / ≥22 | 22 / S | 13 / R | 23 / S |
| **Tetracycline (30 µg)** | ≤11 / ≥15 | 16 / S | 10 / R | 11 / R |
| **Azithromycin (15 µg)** | ≤12 / ≥13 | - | 10 / R | 10 / R |
| **Doxycycline (30 µg)** | ≤10 / ≥14 | - | 15 / S | 12 / R |
| **Vancomycin (30 µg)** | ≤14 / ≥17 | 18 / S | - | - |

1. Humphries, R., Bobenchik, A. M., Hindler, J. A., & Schuetz, A. N. (2021). Overview of changes to the clinical and laboratory standards institute performance standards for antimicrobial susceptibility testing, M100. *Journal of clinical microbiology*, *59*(12), 10-1128.‏
2. Reller, L. B., Weinstein, M., Jorgensen, J. H., & Ferraro, M. J. (2009). Antimicrobial susceptibility testing: a review of general principles and contemporary practices. *Clinical infectious diseases*, *49*(11), 1749-1755.‏

# Table (2):Antibiotic Susceptibility Profile of Clinical Isolates.

| Antibiotic | *Staphylococcus aureus* (clinical) | *Escherichia coli* (clinical) | *Pseudomonas aeruginosa* (clinical) | *Klebsiella pneumoniae* (clinical) |
| --- | --- | --- | --- | --- |
| ESBL | - | - | - | - |
| Ampicillin | R | R | - | R |
| Amikacin | - | R | R | R |
| Gentamicin | R | R | R | R |
| Ciprofloxacin | R | S | R | R |
| Levofloxacin | S | S | R | - |
| Vancomycin | R | - | - | - |
| Erythromycin | R | - | - | - |
| Clindamycin | S | - | - | - |
| Linezolid | R | - | - | - |
| Teicoplanin | R | - | - | - |
| Tigecycline | S | - | - | - |
| Fusidic Acid | R | - | - | - |
| Rifampicin | R | - | - | - |
| Trimethoprim/Sulfamethoxazole | R | R | - | R |
| Meropenem | - | R | R | R |
| Piperacillin/Tazobactam | - | R | R | R |
| Ceftazidime | - | R | R | R |
| Cefepime | - | R | R | R |
| Cefazolin | - | R | R | R |
| Ampicillin/Sulbactam | - | R | R | - |
| Cefoxitin | - | - | R | - |
| Tobramycin | - | - | R | - |
